# Correlation and agreement between arterial and venous blood gas analysis in patients with hypotension—an emergency department-based cross-sectional study

**DOI:** 10.1186/s12245-023-00486-0

**Published:** 2023-03-10

**Authors:** Hari Prasad, Nagasubramanyam Vempalli, Naman Agrawal, U. N. Ajun, Ajmal Salam, Soumya Subhra Datta, Ashutosh Singhal, Nishant Ranjan, P. P. Shabeeba Sherin, G. Sundareshan

**Affiliations:** 1 Department of Emergency Medicine, All India Institute of Medical Sciences Rishikesh, Rishikesh, Uttarakhand 249203 India; 2 Department of Emergency Medicine, All India Institute of Medical Sciences Raipur, Raipur, Chhattisgarh 492099 India; 3 Department of Community and Family Medicine, All India Institute of Medical Sciences Rishikesh, Rishikesh, Uttarakhand 249203 India; 4 Department of Paediatrics, All India Institute of Medical Sciences Rishikesh, Rishikesh, Uttarakhand 249203 India; 5 Department of Emergency Medicine, Tata Motors Hospital, Jamshedpur, 831004 India; 6 Department of Physical Medicine and Resuscitation, All India Institute of Medical Sciences Rishikesh, Rishikesh, Uttarakhand 249203 India; 7 Department of Radiodiagnosis, All India Institute of Medical Sciences Rishikesh, Rishikesh, Uttarakhand 249203 India

**Keywords:** Blood gas analysis, Emergency, Hypotension, Oxygenation, Shock

## Abstract

**Background:**

Blood gas analysis is integral to assessing emergency department (ED) patients with acute respiratory or metabolic disease. Arterial blood gas (ABG) is the gold standard for oxygenation, ventilation, and acid–base status but is painful to obtain. Peripheral venous blood gas (VBG) is a valuable alternative as it is less painful and easy to collect. The comparability of ABG and VBG was studied in various conditions. But in hypotension, previous findings were inconsistent. So, we studied the correlation and agreement between ABG and VBG in hypotensive patients.

**Methodology:**

The study was conducted at the emergency department of a tertiary healthcare center in Northern India. Patients with hypotension above 18 years who satisfied the inclusion criteria were clinically evaluated. Patients who require ABG as a part of routine care were sampled. ABG was collected from the radial artery. VBG was obtained from the cubital or dorsal hand veins. Both samples were collected within 10 min and were analyzed. All ABG and VBG variables were entered in premade proforma. The patient was then treated and disposed of according to institutional protocol.

**Results:**

A total of 250 patients were enrolled. The mean age was 53.25 ± 15.71 years. 56.8% were male. The study included 45.6% septic, 34.4% hypovolemic, 18% cardiogenic, and 2% obstructive shock patients. The study found a strong correlation and agreement for ABG and VBG pH, pCO2, HCO3, lactate, sodium, potassium, chloride, ionized calcium, blood urea nitrogen, base excess, and arterial/alveolar oxygen ratio. Hence, regression equations were made for the aforementioned. There was no correlation observed between ABG and VBG pO2 and SpO2. Our study concluded that VBG could be a reasonable alternative for ABG in hypotensive patients. We can also mathematically predict values of ABG from VBG using regression equations derived.

**Conclusions:**

ABG sampling causes most unpleasant experiences to patients and is associated with complications like arterial injury, thrombosis, air or clotted-blood embolism, arterial occlusion, hematoma, aneurysm formation, and reflex sympathetic dystrophy. The study has shown strong correlations and agreements for most ABG and VBG parameters and can predict ABG mathematically using regression formulas formulated from VBG. This will decrease needle stick injury, consume less time, and make blood gas evaluation easy in hypotensive settings.

## Background

The first examination of gas content in blood dates back to the 1670 s when Magnus, Hooke, and Boyle obtained gas from the blood by employing a vacuum pump [1]. Hasselbalch adapted Henderson’s laws to the logarithmic form in 1917, creating the Henderson-Hasselbalch equation (pH = pK + log [HCO3 −]/[CO2]), which forms the foundation of clinical acid–base analysis [2, 3].

The clinical use of blood gas analysis originated from the poliomyelitis epidemic in the early 1950s, which showed unprecedented mortality rates in Denmark [4].

In emergency departments (ED), blood gas analysis is used for three primary purposes: establishing acid–base state (mainly pH but also to lesser extent bicarbonate and pCO2), assessing ventilation function (mainly pCO2 but also pH and to a lesser extent pO2), and obtaining lactic acid levels in sepsis. These parameters are measured by blood gas analysis. Historically, analyses were performed on arterial blood [5]. Since the original description in the nineteenth century, the techniques for assessing gas tensions in blood have undergone refinements resulting in accurate point-of-care machines. The physiological description of respiratory failure and metabolic status was initially defined in arterial gas tensions and pH measurements. As a result, various clinical guidelines for treating respiratory failure describe using ABG for evaluation and response to treatment [6].

The parameters commonly measured by modern blood gas analyzers include pH; PCO2; partial pressure of O2 (PO2); concentration of hemoglobin (Hb); dyshemoglobin—COHb (carboxyhemoglobin) and MetHb (methemoglobin); lactate; glucose; and electrolytes (sodium, potassium, and chloride). At the same time, HCO3 and base excess are calculated from measured values [7].

Arterial blood gas analysis is the gold standard to obtain information about oxygenation, ventilation, and acid–base status. Parameters like pH, pO2, pCO2, HCO3, lactate, and base excess obtained from ABG are considered the gold standard. Peripheral venous blood gas (VBG) sampling is a valuable alternative to arterial blood gas (ABG) sampling in the emergency department evaluating metabolic and acid–base disorders. It is easier to obtain venous blood, so obtaining venous blood gas (VBG) is less painful, and samples may be drawn along with sampling for other laboratory tests. Venous blood sampling reduces the risk of arterial hematoma, dissection, and thrombosis. So, it is increasingly performed these days in the emergency department [1, 2].

Peripheral, mixed, and central venous blood can also be sampled. Venous blood gas (VBG) measurements obtained from peripheral, mixed, or central venous blood can be used interchangeably with ABGs to assess acid–base status in hemodynamically stable critically ill patients [8]. Adrogue and Weil concluded in their study that in the presence of severe circulatory failure, there is a worse agreement between arterial and central or mixed venous values, with central or mixed venous blood having a higher CO2 concentration and lower pH than arterial blood because of impaired removal of generated CO2 from the tissues. This increase in the venous–arterial PCO2 difference occurs in states of decreased flow irrespective of the reason for the circulatory failure and has an inverse relationship with cardiac output [9].

The main complications of arterial blood gas measurements include arterial injury, thrombosis, air or clotted-blood embolism, arterial occlusion, hematoma, aneurysm formation, and reflex sympathetic dystrophy [10].

Turner et al. evaluated recall of patients’ collective experience in their ICU stay. He found that ABG sampling was rated by 48% of the patients as the most unpleasant experience during admission, followed by tracheal suction in 44% of the patients [11].

Till 2006, only 22 cases of radial artery aneurysms were reported in the literature [12]. Less severe adverse events such as hematoma after radial artery puncture occur in up to 59% of the patients [13].

Many studies have found a strong correlation between arterial pH, partial pressure of CO2 (PCO2), and calculated bicarbonate and corresponding venous values in different clinical conditions [14]. In patients with diabetic ketoacidosis, pH and PCO2 levels obtained from venous blood gas reasonably correlate with ABG values [15]. Elborn et al. conducted a study on COPD patients and found no significant difference between the arterial and venous CO2 tensions, and the two were closely correlated [16]. In a study by Rees et al. on 40 patients with chronic lung disease, they supported the use of peripheral VBG to estimate PaO2 in a vast majority of patients. They also showed that peripheral venous carbon dioxide tension and pH correlate well with arterial values [17]. In another study, Malinoski et al. concluded that VBG and arterial PCO2, pH, and base excess values had a good correlation [18].

Hypotension is one condition where findings of ABG and VBG comparability are inconsistent. Few studies tried to study the correlation between arterial and venous samples in patients with hypotension, but their study findings are not uniform and vary widely. The various studies done on comparability between ABG and VBG are shown in Table 1.

**Table 1:** Various studies done on comparability between ABG and VBG

Study name	Study setting	Parameters that showed correlation	Parameters that did not show correlation	Year of publication
Yildizdas et al. [19]	ICU	pH, pCO2, HCO3, base excess	pO2	2004
Malinoski et al. [18]	ICU	pH, pCO2, and base excess	pO2	2005
Kelly et al. [20]		HCO3	pCO2	2010
Shirani et al. [21]	ED	pH, HCO3, base excess	pCO2	2011
Kim et al. [22]	ICU	pH, pCO2, HCO3	-	2013
Byrne et al. [23]		pH	pCO2	2014
Hynes et al. [24]	ICU	pH, HCO3, lactate, base excess	-	2015
Zeserson et al. [25]	ED, ICU	pH, pCO2	-	2018
White et al. [26]	ICU	pH	pCO2, HCO3	2018
Rudkin et al. [27]	ED	-	pH, pCO2	2020
Shin et al. [28]	ED	pH, HCO3, Ca2 + , Na + , K + , Cl − , lactate, glucose	pCO2	2020
Nanjayya et al. [29]	ICU	pH	pCO2	2020
Boon et al. [30]	ED	Lactate	pH, base deficit	2021

ABG sampling is technically challenging and time-consuming, is necessitating sampling skill, and is occasionally associated by the risk of staff needle stick exposure. Because venous blood sample requires fewer punctures, the danger of needle stick injury in medical staff is reduced [21].

Therefore, we intend to do correlation and agreement between ABG and VBG in hypotensive patients. Also, there were no studies done correlating values of ABG and VBG in hypotensive patients in academic emergency medicine settings in India to our knowledge.

Our cross-sectional study aimed to determine the relationship and agreement between arterial and venous blood gas analysis for the parameters pH, pCO2, HCO3, lactate, and base excess in hypotensive patients in the emergency department and also to develop prediction models in measuring parameters like pH, pCO2, HCO3, lactate, and base excess in arterial and venous samples.

## Methods

### Study design and settings

This cross-sectional study was conducted in the Emergency Department of All India Institute of Medical Sciences, Rishikesh, Uttarakhand, between January 2021 and June 2022 (patient recruitment period from March 2021 to March 2022).

### Selection of patients

#### Inclusion criteria

1. Patients with systolic blood pressure less than 90 mmHg or MAP less than 65 mmHg in the emergency department who require ABG as a part of routine care
2. Patients more than 18 years of age

#### Exclusion criteria

1. Those who did not give consent
2. Existence of contraindications for arterial blood sampling, including impalpable or negative Allen’s test in the upper extremities, infection or fistula at the desired site of puncture, or having severe coagulation disorders
3. Interval of more than 10 min between arterial and venous sampling and inappropriate sample transfer to the laboratory
4. Postcardiac arrest patients

### Sample size

Patients with hypotension above 18 years of age in the emergency department who satisfied the inclusion criteria were clinically evaluated. The minimum sample size was found to be 14 [22]. Because of the very small sample size, we included as much as participants using the consecutive sampling method during the study course (a total of 250 patients were included).

### Operational definition of hypotension

Patients with systolic blood pressure less than 90 mmHg or mean arterial blood pressure less than 65 mmHg.

### Clinical evaluation

Clinical evaluations were performed on emergency room patients with hypotension who were older than 18 years and met the inclusion criteria. After explaining the study to the patient or relative, written consent was obtained. The patient’s demographic details, vitals, clinical details, and diagnosis were entered in the data collection proforma. Patients who required ABG as a part of routine care per treating physician were sampled. An arterial sample (0.5–1 mL) was collected using a heparinized syringe from the radial artery at the wrist level. The venous blood sample was obtained from the cubital or dorsal hand veins. Both samples were collected with minimum delay (less than 10 min). Both samples were analyzed as soon as possible using a blood gas analyzer Nova Biomedicals Stat profile pHOX ultra.

### Data collection

**Fig. 1:**
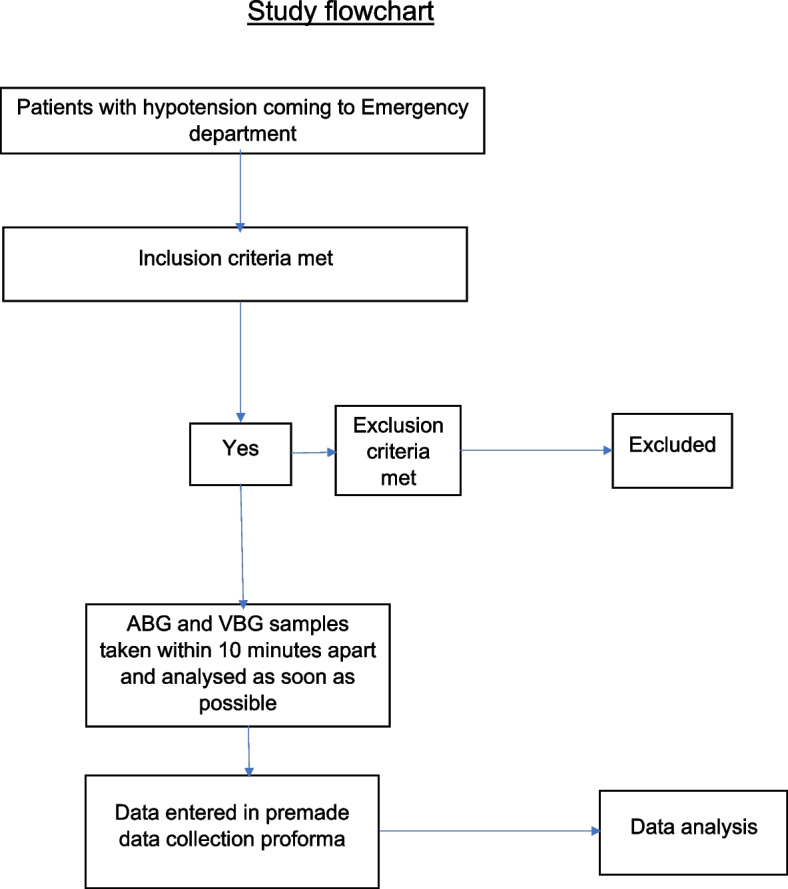
Flow chart

All variables of ABG and VBG were entered in premade data collection proforma. The patient was then treated and disposed of according to institutional protocol. The study flowchart is attached (Fig. 1).

### Statistical analysis

All data were entered into an Excel sheet and analyzed using SPSS 23.0 version. Descriptive statistics for numerical variables were calculated as the mean and standard deviation for normal distribution and median (IQR) for non-normal distribution, whereas percentages for qualitative variables. Agreement between arterial and venous blood gas analysis parameters was done using the Bland–Altman plot. Pearson correlation coefficients were applied to estimate the correlation between arterial and venous blood gas analysis parameters for Gaussian distribution and the Spearman correlation coefficient for non-Gaussian distribution. Arterial parameters were predicted from venous samples using linear regression.

## Results

Clinical evaluations were performed on emergency room patients with hypotension who were older than 18 and met the aforementioned inclusion criteria. We obtained their consent and enrolled them in our research. Of all the patients, 250 patients were finally analyzed in the study.

### Baseline characteristics of the study population

The study population’s main baseline characteristics and comorbidities are shown in Table 2.

**Table 2:** Baseline characteristics of the study population

**Characteristics**	**Values**
Age in years, mean (standard deviation)	53.25 ± 15.71
Men, number (%)	142 (56.8%)
Women, number (%)	108 (43.2%)
**Final diagnosis**	**Number (%)**
Infectious diseases	27 (10.8%)
Cerebrovascular accident	11 (4.4%)
Acute gastroenteritis	9 (3.6%)
Acute decompensated heart failure	3 (1.2%)
Poisoning	7 (2.8%)
Malignancy	24 (9.6%)
Pneumonia	29 (11.6%)
Chronic liver disease	22 (8.8%)
Intestinal obstruction	3 (1.2%)
Pulmonary thromboembolism	2 (0.8%)
Cardiac tamponade	2 (0.8%)
Acute on chronic kidney disease	5 (2.0%)
Acute pancreatitis	2 (0.8%)
Obstructive airway disease	19 (7.6%)
Acute coronary syndrome	17 (6.8%)
Diabetic ketoacidosis	3 (1.2%)
Dilated cardiomyopathy	7 (2.8%)
Trauma	3 (1.2%)
Urosepsis	8 (3.2%)
Others	47 (18.8%)
**Type of shock**	**Number (%)**
Septic	114 (45.6%)
Hypovolemic	86 (34.4%)
Cardiogenic	45 (18.0%)
Obstructive	5 (2.0%)

### Examination findings

The main examination findings are shown in Table 3.

**Table 3:** Examination findings

**Examination**	**Values**
Pulse rate in beats/minute, mean (standard deviation)	103.78 ± 16.52
Respiratory rate in breaths/minute, mean (standard deviation)	21.67 ± 4.31
Systolic BP in mmHg, mean (standard deviation)	81.40 ± 6.01
Diastolic BP in mmHg, mean (standard deviation)	49.31 ± 4.42
MAP in mmHg, mean (standard deviation)	59.98 ± 4.16
**SpO2 to maintain target saturation of 94% or more except 88–92% for obstructive airway disease**	**Number (%)**
Room air	174 (69.6%)
Oxygen with nasal prongs	41 (16.4%)
Oxygen with face mask	18 (7.2%)
Oxygen with non-rebreather mask	1 (0.4%)
NIV	4 (1.6%)
Ventilator	12 (4.8%)
Temperature in F, mean (standard deviation)	98.68 ± 0.44
GCS, mean (standard deviation)	13.75 ± 3.11
**POCUS findings**	**Values**
Lung profile	**Number (%)**
A profile	123 (49.2%)
B profile	127 (50.8%)
Lung sliding (yes)	249 (99.6%)
Effusion (yes)	11 (4.4%)
Other findings	
None	238 (95.2%)
Mild pleural effusion	7 (2.8%)
Moderate pleural effusion	3 (1.2%)
Gross pleural effusion	1 (0.4%)
Mass lesion	1 (0.4%)
Contractility	
Fair	210 (84.0%)
Moderately reduced	27 (10.8%)
Severely reduced	13 (5.2%)
Ejection fraction, mean (standard deviation)	51.52 ± 8.73
IVC size (cm), mean (standard deviation)	1.53 ± 0.34

### Assessment of parameters

The assessment of various parameters of ABG and VBG with mean, standard difference, and *p*-value is shown in Table 4.

**Table 4:** Statistically significant correlation between ABG and VBG

Parameters	ABG (mean ± SD)	VBG (mean ± SD)	Absolute difference (mean ± SD)	*P*-value
pH	7.37 (0.11)	7.34 (0.12)	− 0.03 (0.03)	< 0.001
pO2 (mmHg)	87.76 (20.78)	37.60 (12.80)	− 50.17 (21.81)	< 0.001
pCO2 (mmHg)	32.76 (15.25)	36.17 (16.02)	3.41 (5.19)	< 0.001
HCO3 (mmol/L)	18.57 (6.11)	19.09 (6.27)	0.52 (1.53)	< 0.001
Lactate (mmol/L)	2.84 (2.70)	2.97 (2.83)	0.13 (0.82)	< 0.001
Sodium (mmol/L)	139.09 (8.52)	138.97 (8.77)	− 0.12 (3.40)	0.365
Potassium (mmol/L)	3.91 (0.71)	3.92 (0.71)	0.02 (0.31)	0.114
Chloride (mmol/L)	108.79 (7.93)	108.70 (7.88)	− 0.10 (2.81)	0.590
Ionized calcium (mmol/L)	0.93 (0.20)	0.91 (0.19)	− 0.01 (0.15)	0.350
BUN (mg/dL)	35.89 (22.15)	35.73 (21.90)	− 0.16 (4.89)	0.432
Base excess (mmol/L)	− 5.41 (5.78)	− 5.12 (5.90)	0.29 (1.45)	0.001
Arterial/alveolar oxygen ratio (a/A)	0.87 (0.29)	0.37 (0.41)	− 0.50 (0.26)	< 0.001
Oxygen saturation (%)	95.93 (2.68)	59.90 (19.11)	− 36.03 (19.16)	< 0.001

### Assessment of correlation, agreement, and regression of various parameters

**Fig. 2:**
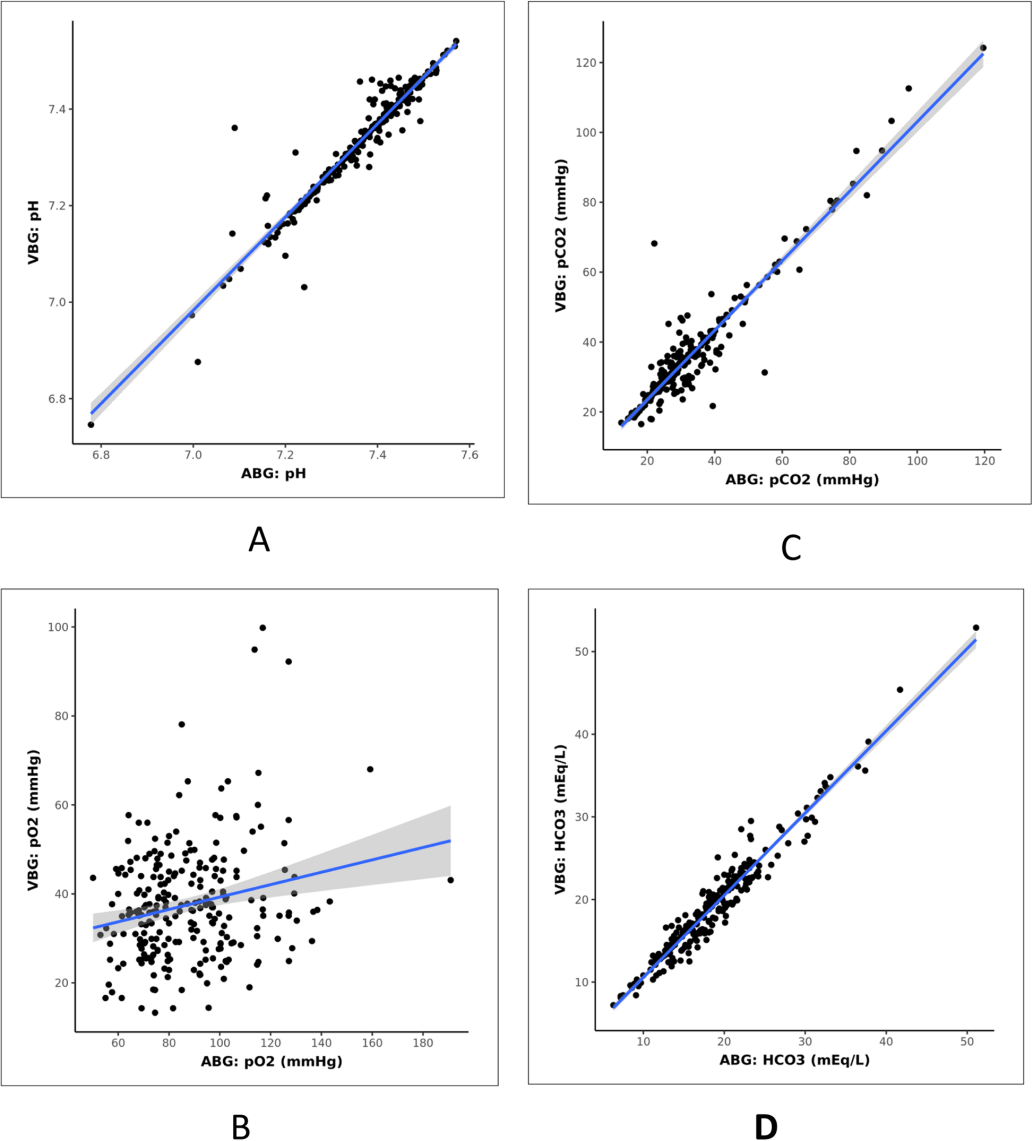
Scatterplot for correlation between ABG and VBG for various parameters: **A** pH, **B** pO2, **C** pCO2, and **D** HCO3

**Fig. 3:**
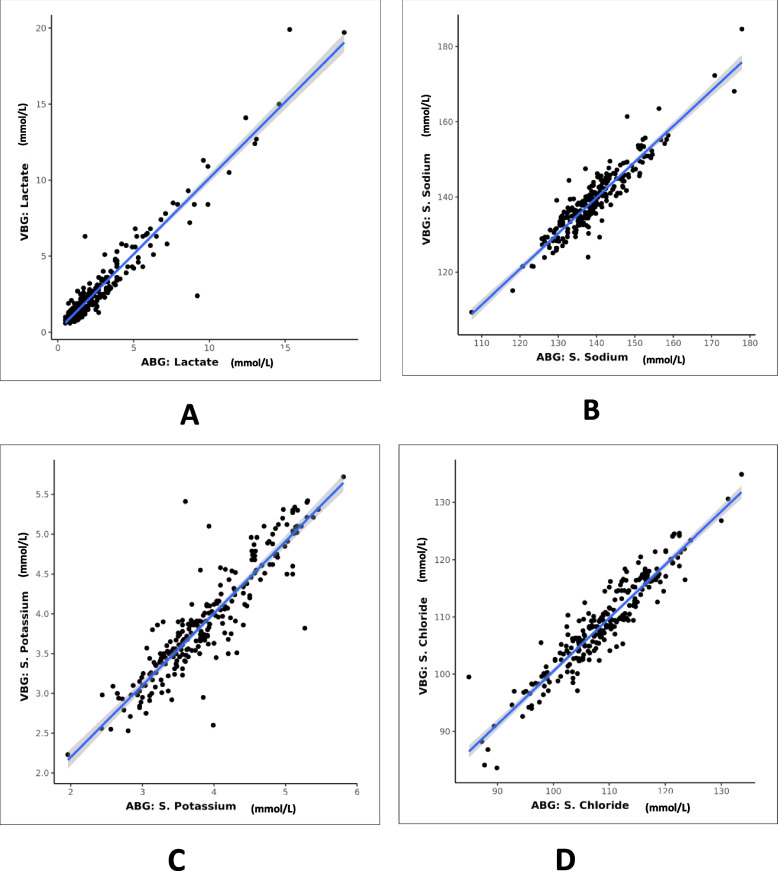
Scatterplot for correlation between ABG and VBG for various parameters: **A** lactate, **B** sodium, **C** potassium, and **D** chloride

**Fig. 4:**
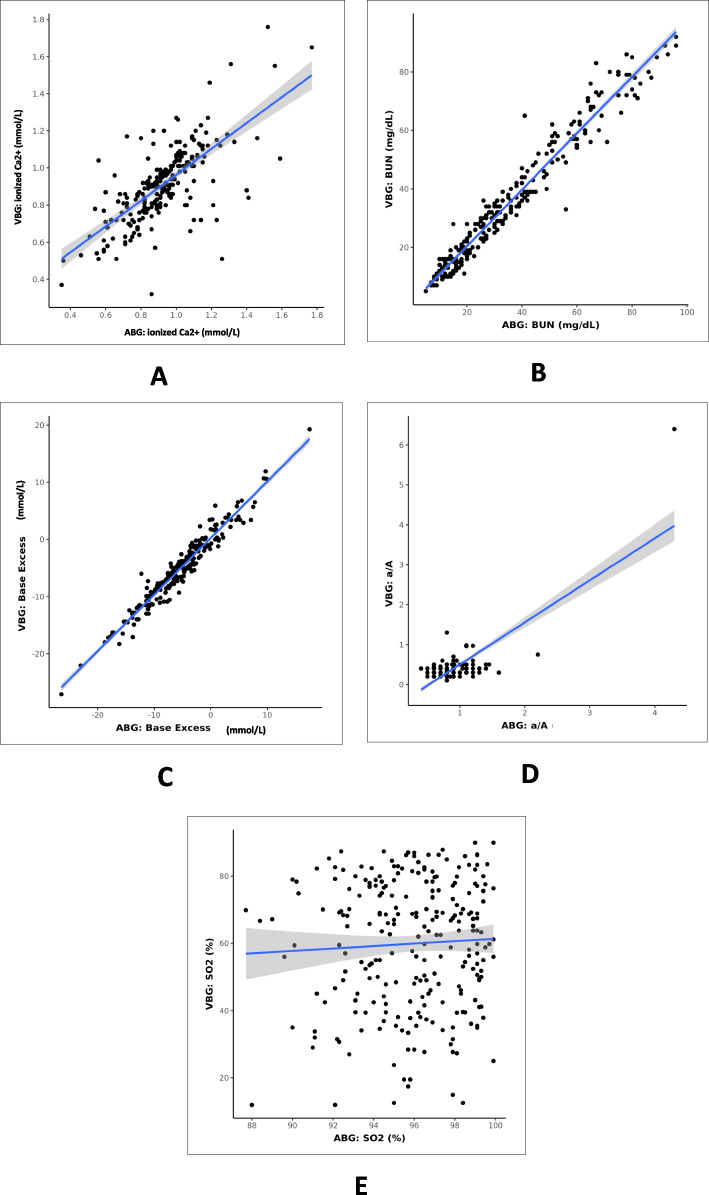
Scatterplot for correlation between ABG and VBG for various parameters: **A** ionized calcium, **B** BUN, **C** base excess, **D** a/A, and **E** SO2

**Fig. 5:**
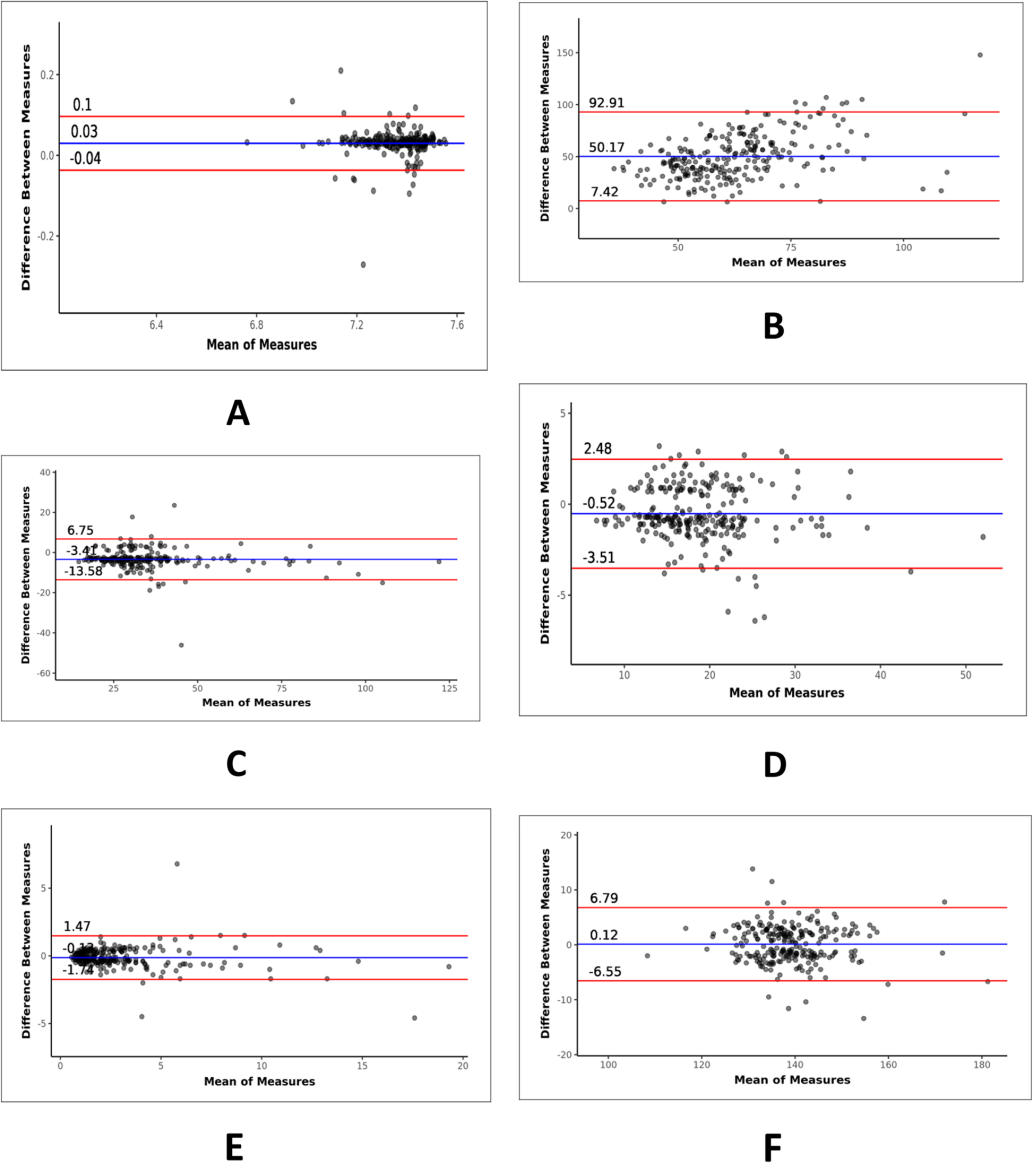
Bland–Altman plot for agreement between ABG and VBG for various parameters: **A** pH, **B** pO2, **C** pCO2, **D** HCO3, **E** lactate, and **F** sodium

**Fig. 6:**
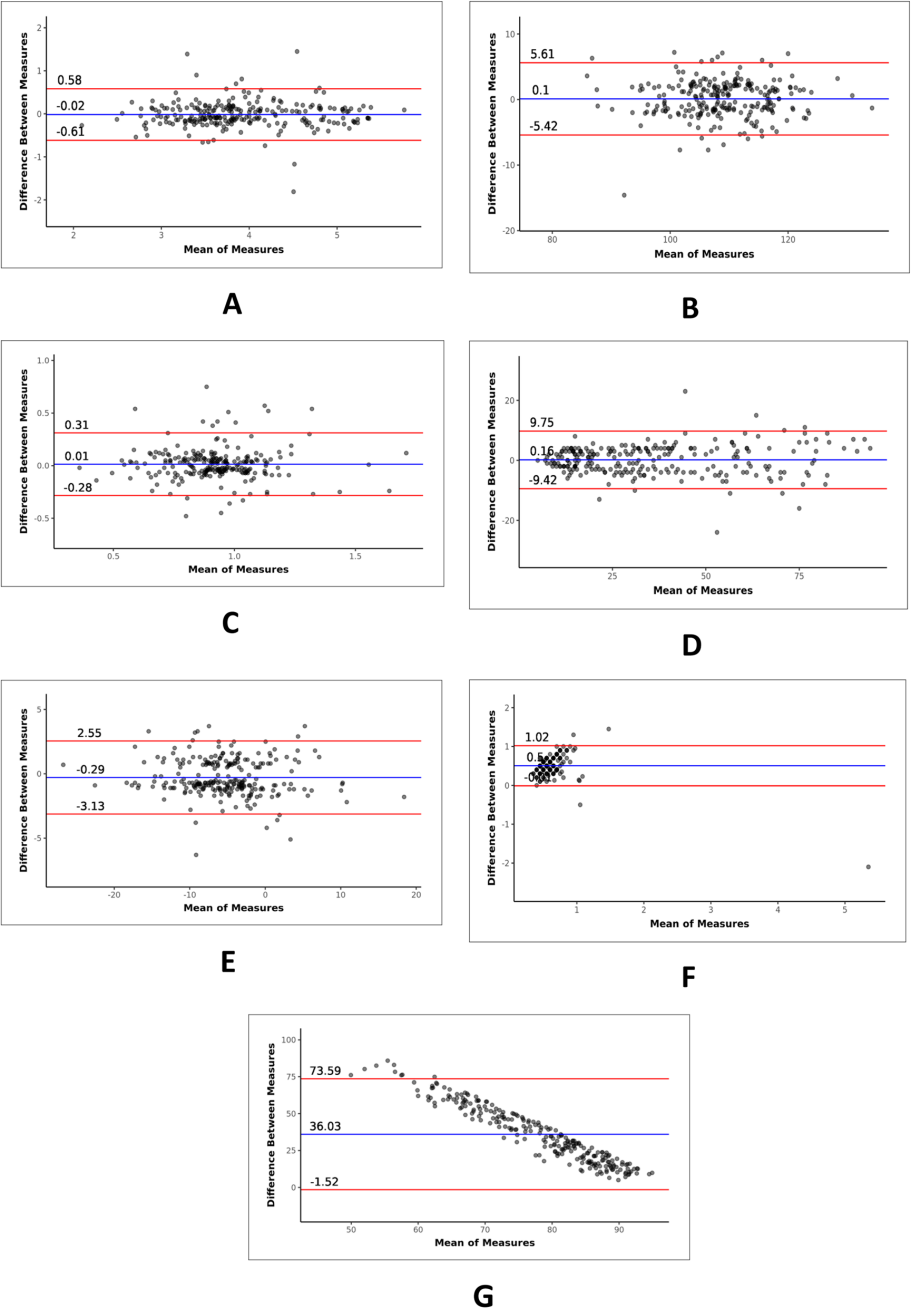
Bland–Altman plot for agreement between ABG and VBG for various parameters: **A** potassium, **B** chloride, **C** ionized calcium, **D** BUN, **E** base excess, **F** a/A, and **G** SO2

Correlation and agreement of various parameters studied are shown in Table 5. Scatterplots showing the correlation of various parameters are shown in Figs. 2, 3, and 4. Bland–Altman plots showing agreement of various parameters are shown in Figs. 5 and 6. Regression analysis done for parameters shown strong correlation and agreement are as follows:

**Table 5:** Correlation and agreement of various parameters

Parameters	Interclass correlation coefficient	Limits of agreement
pH	0.96	± 0.07
pO2 (mmHg)	0.20	± 42.74
pCO2 (mmHg)	0.95	± 10.16
HCO3 (mmol/L)	0.97	± 3.00
Lactate (mmol/L)	0.96	± 1.61
Sodium (mmol/L)	0.92	± 6.67
Potassium (mmol/L)	0.91	± 0.60
Chloride (mmol/L)	0.94	± 5.51
Ionized calcium (mmol/L)	0.70	± 0.30
BUN (mg/dL)	0.98	± 9.59
Base excess (mmol/L)	0.97	± 2.84
Arterial/alveolar oxygen ratio (a/A)	0.72	± 0.52
Oxygen saturation (%)	0.01	± 37.55

$$A\mathrm{BG}:\mathrm{pH}=0.42+0.95\times\mathrm{VBG}:\mathrm{pH}(R^2=0.91)$$

$$\mathrm{ABG}:\mathrm{pCO}2 (\mathrm{mmHg}) = 0.19 + 0.9 \times \mathrm{ VBG}:\mathrm{pCO}2 (\mathrm{mmHg}) ({R}^{2}=0.90)$$

$$A\mathrm{BG}:\mathrm{HCO}3 (\mathrm{mmol}/\mathrm{L}) = 0.53 + 0.95 \times \mathrm{ VBG}:\mathrm{HCO}3 (\mathrm{mmol}/\mathrm{L}) ({R}^{2}=0.94)$$

$$\mathrm{ABG}:\mathrm{lactate }(\mathrm{mmol}/\mathrm{L}) = 0.12 + 0.92 \times \mathrm{ VBG}:\mathrm{lactate }(\mathrm{mmol}/\mathrm{L}) ({R}^{2}=0.92)$$

$$\mathrm{ABG}:\mathrm{S}.\mathrm{ sodium }(\mathrm{mmol}/\mathrm{L}) = 14.44 + 0.9 \times \mathrm{ VBG}:\mathrm{S}.\mathrm{ sodium }(\mathrm{mmol}/\mathrm{L}) ({R}^{2}=0.85)$$

$$\mathrm{ABG}:\mathrm{S}.\mathrm{ potassium }(\mathrm{mmol}/\mathrm{L}) = 0.34 + 0.91 \times \mathrm{ VBG}:\mathrm{S}.\mathrm{ potassium }(\mathrm{mmol}/\mathrm{L}) ({R}^{2}= 0.82)$$

$$\mathrm{ABG}:\mathrm{S}.\mathrm{ chloride }(\mathrm{mmol}/\mathrm{L}) = 6.3 + 0.94 \times \mathrm{ VBG}:\mathrm{S}.\mathrm{ chloride }(\mathrm{mmol}/\mathrm{L}) ({R}^{2}=0.88)$$

$$\mathrm{ABG}:\mathrm{ionized Ca}2+ (\mathrm{mmol}/\mathrm{L}) = 0.29 + 0.7 \times \mathrm{ VBG}:\mathrm{ionized Ca}2+ (\mathrm{mmol}/\mathrm{L}) ({R}^{2}=0.49)$$

$$\mathrm{ABG}:\mathrm{BUN }(\mathrm{mg}/\mathrm{dL}) = 0.65 + 0.99 \times \mathrm{ VBG}:\mathrm{BUN }(\mathrm{mg}/\mathrm{dL}) ({R}^{2}=0.95)$$

$$\mathrm{ABG}:\mathrm{ Base E}\times \mathrm{cess }(\mathrm{mmol}/\mathrm{L}) = -0.55 + 0.95 \times \mathrm{ VBG}:\mathrm{base excess }(\mathrm{mmol}/\mathrm{L}) ({R}^{2}=0.94)$$

$$\mathrm{ABG}:\mathrm a/\mathrm A=0.67+0.55\times\mathrm{VBG}:\mathrm a/\mathrm A(R^{\mathit2}=0.58)$$

## Discussion

Our study enrolled 250 patients with hypotension and required ABG as a part of routine care and satisfied inclusion criteria.

### Demographics

Our study population comprised 250 patients with a mean (standard deviation) years of 53.25 (± 15.71). The study included 56.8% (142) males and 43.2% (108) females. A study by Kim et al. done on 34 patients had a mean age of 65.57 ± 12.4 years, and males were 58.8% (20) and females 41.2% (14) [22]. In a study done by Shirani et al., 192 patients had a mean age of 51.6 ± 23.6 years, and there were males 67.7% and females 32.3% [21], whereas in the study by Shin et al., 231 hypotensive patients had a mean age (SD) of 68.2 (16.6) years and males 64.5% and females 35.5% [28].

50.8% were residents of Uttarakhand, 46.8% were residents of Uttar Pradesh, and 2.4% were from other states. 45.6% of patients were septic, 34.4% hypovolemic, 18% cardiogenic, and 2% obstructive shock.

### Size and site of needle used

For ABG, 24G needles were used in all 250 patients, whereas for VBG, 18G was used for sampling in 59.6% of cases, and in 40.4% of cases, a 20G needle was used. The radial artery obtained all ABGs in our study, while VBG was obtained from forearm veins. In the study by White et al., ABG was collected by either needle puncture or withdrawal from an arterial catheter. The VBG was collected from the upper extremity, and a tourniquet could be left in place for no more than 1 min [26].

### pH

There was a very strong correlation between ABG:pH and VBG:pH, and this correlation was statistically significant (interclass correlation coefficient = 0.96, *p* ≤ 0.001) in our study, with 94.0% of the observations having a difference which was within the limits of agreement (± 0.07). The *r*-value was 0.91 and the formula for ABG pH was devised as ABG:pH = 0.42 + 0.95 × VBG:pH. In the study by Yildizdas et al., pH showed a good interclass correlation of 0.907 with a *p*-value < 0.001. The *r*-value for pH for that study was 0.994 [19]. In the study by White et al., pH had an interclass correlation of 0.90 with a *p*-value < 0.001. Bias ± SD of ABG-VBG was 0.03 ± 0.04 [26]. In Zeserson et al. study, the mean difference for pH between VBG and ABG was 0.03 (95% confidence interval: 0.03–0.04) with a Pearson correlation of 0.94 [25]. In the study by Nanjayya et al., the mean bias for pH was + 0.036 with 95% LOA ranging from − 0.005 to + 0.078 [29]. In a meta-analysis by Byrne et al., there was little difference between the pH obtained from the PVBG and the ABG, with the arterial pH typically 0.03 higher than the venous pH (95% confidence interval 0.029–0.038) [23]. Kim et al. found a good correlation between ABG and VBG pH with a Pearson correlation coefficient of 0.783 with a *p*-value of 0.0001. Regression equations were derived to predict ABG pH from peripheral VBG pH as follows: arterial pH = 0.763 × venous pH + 1.786 (*R* ^2^ = 0.544). The multivariate regression equations were found as follows: arterial pH =  − 1.108 + 1.145 × venous pH + 0.008 × PCO2 – 0.012 × venous HCO3 + 0.002 × venous total CO2 (*r* = 0.655) [22]. In a study by Shirani et al., the average VBG-ABG amount of difference (95% limits of agreement) in the hypotension versus normotensive group were − 0.030 (− 0.09 to 0.03) vs. − 0.016 (− 0.1 to 0.068) for pH (*p* = 0.01) [21]. In studies by Boon et al. and Rudkin et al., pH was not correlating between ABG and VBG [27, 30].

### pO2

There was a weak correlation between ABG:pO2 (mmHg) and VBG:pO2 (mmHg), and this correlation was statistically significant (interclass correlation coefficient = 0.20, *p* ≤ 0.001 in our study). No correlation was found between ABG:pO2 and VBG:pO2 in previous studies.

### pCO2

There was a very strong correlation between ABG:pCO2 (mmHg) and VBG:pCO2 (mmHg), and this correlation was statistically significant (interclass correlation coefficient = 0.95, *p* =  < 0.001) in our study. In the study by Yildizdas et al., there was a good correlation between ABG and VBG with an interclass correlation of 0.978 with a *p*-value < 0.001. The *r*-value found was 0.957 [19]. In the study by Malinoski et al., pCO2 values have a good correlation and agreement between ABG and VBG values with *R* = 0.88, *p* < 0.001, and 95% LOAs of − 2.2 to 10.9 [18]. Kim et al., in their study, found a good Pearson correlation coefficient of 0.705 with a *p*-value of 0.0001 for pCO2. Regression equation derived for pCO2-arterial PCO2 = 0.611 × venous PCO2 + 9.521 (*R* ^2^ = 0.497). Multivariate regression equation derived was arterial PCO2 = 88.6 − 10.888 × venous pH + 0.150 × PCO2 + 0.812 × venous HCO3 + 0.124 × venous total CO2 (*r* = 0.609) [22], whereas in the study by Zeserson et al., the mean difference for pCO2 between VBG and ABG was 4.8 mm Hg (95% confidence interval: 3.7–6.0 mm Hg) with a Pearson correlation of 0.93 [25]. In our study, 95.6% of the observations had a difference within the limits of agreement (± 10.16). *R* ^2^ = 0.90 for pCO2 (mmHg) and the formula was devised as ABG:pCO2 (mmHg) = 0.19 + 0.9 × VBG:pCO2 (mmHg).

### HCO3

There was a very strong correlation between ABG:HCO3 (mmol/L) and VBG:HCO3 (mmol/L), and this correlation was statistically significant (interclass correlation coefficient = 0.97, *p* ≤ 0.001) in our study. In the study by Shin et al., there was a good correlation and agreement between ABG and VBG HCO3 with a mean (SD) of 19.27 (5.39) with 95% CI as − 1.927 to − 1.461 and LOA ± 3.53 [28]. Yildizdas et al. found that HCO3 has a good Pearson correlation coefficient of 0.976 with a *p*-value < 0.001 and *r* = 1.676 [19]. Venous–arterial difference of HCO3 − is − 0.37mmmol/L with good agreement in the study by Hynes et al. [24]. Pearson correlation coefficient of HCO3 in a study by Kim et al. was 0.846 with a *p*-value of 0.0001. The regression equation derived for HCO3 was arterial HCO3 = 0.822 × venous HCO3 + 2.815 (*R* ^2^ = 0.716). Multivariate regression equation derived was arterial HCO3 =  − 89.266 + 12.677 × venous pH + 0.042 × PCO2 + 0.675 × venous HCO3 + 0.185 × venous total CO2 (*r* = 0.782) [22]. In a meta-analysis by Kelly et al., the weighted mean difference between arterial and venous values for bicarbonate was –1.41 mmol/L (*n* = 905), with 95% limits of agreement of the order of ± 5 mmol/L [20]. Shirani et al. found the average VBG-ABG amount of difference (95% limits of agreement) in the hypotension versus normotensive group was 1.79 (− 1.91 to 5.49) vs. 1.32 (− 1.94 to 4.58) mEq/L for HCO3 (*p* = 0.032) [21]. In our study, 93.6% of the observations had a difference within the limits of agreement (± 3.00). *R* ^2^ = 0.94 for HCO3 (mmol/L) and the formula was devised as ABG:HCO3 (mmol/L) = 0.53 + 0.95 × VBG:HCO3 (mmol/L)*.*

### Lactate

There was a very strong correlation between ABG:lactate (mmol/L) and VBG:lactate (mmol/L), which was statistically significant (interclass correlation coefficient = 0.96, *p* =  < 0.001) in our study. 97.6% of the observations had a difference within the limits of agreement (± 1.61). *R* ^2^ = 0.92 for lactate (mmol/L) and the formula was devised as ABG:lactate (mmol/L) = 0.12 + 0.92 × VBG:lactate (mmol/L). In a study by Hynes et al., the venous–arterial difference for lactate was found to be 0.16 mmol/L [24]. In a study by Boon et al., venous lactate was clinically equivalent based on the pre-determined threshold limits of − 1.5 to 1.5 mmol/L, where 96.0% of the values were within this acceptable range [30]. Shin et al., in their study, found the mean (SD) for lactate as 3.27 (3.23) with 95% CI (− 0.406, − 0.241) and LOA ± 1.25 [28].

### Sodium

In the study by Shin et al., the mean (SD) for sodium was 136.21 (6.60) with 95% CI (− 3.104, − 2.532) and LOA ± 4.33 [28]. There was a very strong correlation between ABG and VBG S. Sodium (mmol/L), and this correlation was statistically significant (interclass correlation coefficient = 0.92, *p* ≤ 0.001) in our study. 95.6% of the observations had a difference within the limits of agreement (± 6.67). *R* ^2^ = 0.85 for sodium (mmol/L) and the formula was devised as ABG:S. sodium (mmol/L) = 14.44 + 0.9 × VBG:S. sodium (mmol/L).

### Potassium

There was a very strong correlation between ABG and VBG potassium (mmol/L), and this correlation was statistically significant (interclass correlation coefficient = 0.91, *p* ≤ 0.001) in our study. 95.6% of the observations had a difference within the limits of agreement (± 0.60). The regression equation for potassium (mmol/L) was devised as ABG:S. potassium (mmol/L) = 0.34 + 0.91 × VBG:S. potassium (mmol/L) [*R* ^2^ = 0.82], whereas in the study by Shin et al., mean (SD) for potassium was 4.42 (1.18) with 95% CI (− 0.365, − 0.257) and LOA ± 0.82 [28].

### Chloride

The study by Shin et al. found the mean (SD) for chloride as 102.94 (6.31) with 95% CI (− 4.062, − 2.934) and LOA as ± 8.53 [28], whereas in our study, there was a very strong correlation between ABG and VBG chloride (mmol/L), and this correlation was statistically significant (interclass correlation coefficient = 0.94, *p* ≤ 0.001). 94.0% of the observations had a difference within the limits of agreement (± 5.51). The regression equation for chloride was done using *R*^2^ = 0.88 formula was devised as ABG:S. chloride (mmol/L) = 6.3 + 0.94 × VBG:S. chloride (mmol/L). No other studies have done a correlation between ABG and VBG for chloride.

### Ionized calcium

Ionized calcium was found to have a strong correlation between ABG and VBG with an interclass correlation coefficient = 0.70 with *p*-value ≤ 0.001. 94.0% of the observations had a difference which was within the limits of agreement (± 0.30). The regression equation devised for ionized calcium (mmol/L) was ABG:ionized Ca2 + (mmol/L) = 0.29 + 0.7 × VBG:ionized Ca2 + (mmol/L) [*R* ^2^ = 0.49] in our study, whereas in the study by Shin et al., the mean (SD) for ionized calcium was 1.21 (0.08) with 95% CI (0.064, 0.089) and LOA as ± 0.18 [28].

### Blood urea nitrogen

Blood urea nitrogen was found to have a very strong correlation between ABG and VBG with an interclass correlation coefficient = 0.98 and *p*-value ≤ 0.001. 96.0% of the observations had a difference which was within the limits of agreement (± 9.59). The regression equation derived for BUN (mg/dL) was ABG:BUN (mg/dL) = 0.65 + 0.99 × VBG:BUN (mg/dL) [*R* ^2^ = 0.95] in our study. We found no other study which correlates ABG:BUN and VBG:BUN.

### Base excess

There was a very strong correlation between ABG:base excess (mmol/L) and VBG:base excess (mmol/L) with an interclass correlation coefficient = 0.97 and *p* ≤ 0.001 in our study. 94.8% of the observations had a difference within the limits of agreement (± 2.84). The regression equation derived for base excess (mmol/L) was ABG:base excess (mmol/L) =  − 0.55 + 0.95 × VBG:base excess (mmol/L) [*R* ^2^ = 0.94]. The study by Yildizdas et al. found a good correlation with a Pearson correlation coefficient of 0.972 with a *p*-value < 0.001. *R* ^2^ was found to have 0.945 for base excess [19]. Malinoski et al. compared ABG and VBg values and found that base excess has *R* = 0.96, *p* < 0.001, and 95% LOAs of − 2.2 to 1.8 in their study [18]. The average VBG-ABG amount of difference (95% limits of agreement) in the hypotension versus normotensive group found in a study by Shirani et al. was *d* 0.25 (− 3.73 to 4.23) vs. 0.79 (− 2.51 to 4.09) for BE (*p* = 0.036) [21]. In a study by Hynes et al., the venous–arterial difference for base excess was found to be 0.08 mEq/L [24].

### Arterial/alveolar oxygen ratio

Our study found a strong correlation between ABG:a/A and VBG:a/A, and this correlation was statistically significant (interclass correlation coefficient = 0.72, *p* ≤ 0.001). 98.4% of the observations had a difference within the limits of agreement (± 0.52). The regression equation was derived with *R* ^2^ = 0.58 for a/A as ABG:a/A = 0.67 + 0.55 × VBG:a/A. Our study is the first to evaluate and find a correlation for arterial/alveolar oxygen ratio between ABG and VBG.

### Oxygen saturation

Our study found a weak correlation between ABG:SO2 (%) and VBG:SO2 (%), and this correlation was not statistically significant (interclass correlation coefficient = 0.01, *p* = 0.412). All other studies comparing ABG and VBG found weak or no correlation for oxygen saturation.

### Limitations

In our study, patients were recruited using convenience sampling as study investigators will not be available in all shifts. The study involved collecting single pair of arterial and peripheral venous samples from each patient. So, the homogeneity of ABG and peripheral VBG was not studied as multiple samples at different intervals were not done. We also had many cases where one patient had multiple diagnoses making it challenging to characterize into various subgroups. Subgroups of shock were defined at the time of “working diagnosis” when enrolling the patient and not the final diagnosis. The study design and setting did not allow for a follow-up on the mortality of the patients studied. Patients differed according to many demographic factors, which were not all the same.

## Conclusions

ABG and VBG assessments are essential tests for assessing ventilation, acid–base disturbances, and other metabolic parameters of patients. Obtaining ABG or VBG in hypotension is challenging as many previous studies have shown conflicting results. Our study has shown either strong or very strong correlations and agreements for most ABG and VBG parameters except pO2 and SO2. We can also predict an ABG mathematically using regression formulas devised from a VBG sample. This will decrease needle stick injury, consume less time, and make blood gas evaluation easy in hypotensive settings. Further studies are required to find the correlation between ABG and central, peripheral, and mixed VBGs and capillary blood gas. Our study is the first to incorporate all four types of shock in ABG and VBG analysis. Also, this is the first one to incorporate all the parameters of ABG and VBG. Also, this is the largest single-center study in ABG and VBG comparison on hypotensive patients. Our study is the first to incorporate details of the point-of-care ultrasound in hypotensive ABG v VBG studies. We have a standardized site and size of the needle to be used for ABG and VBG in this study.

## Data Availability

The datasets used and/or analyzed during the current study are available from the corresponding author on reasonable request.
